# Non-redundant roles in sister chromatid cohesion of the DNA helicase DDX11 and the SMC3 acetyl transferases ESCO1 and ESCO2

**DOI:** 10.1371/journal.pone.0220348

**Published:** 2020-01-14

**Authors:** Atiq Faramarz, Jesper A. Balk, Janne J. M. van Schie, Anneke B. Oostra, Cherien A. Ghandour, Martin A. Rooimans, Rob M. F. Wolthuis, Job de Lange

**Affiliations:** Cancer Center Amsterdam, Department of Clinical Genetics, section Oncogenetics, Amsterdam University Medical Centers, Amsterdam, the Netherlands; Virginia Tech, UNITED STATES

## Abstract

In a process linked to DNA replication, duplicated chromosomes are entrapped in large, circular cohesin complexes and functional sister chromatid cohesion (SCC) is established by acetylation of the SMC3 cohesin subunit. Roberts Syndrome (RBS) and Warsaw Breakage Syndrome (WABS) are rare human developmental syndromes that are characterized by defective SCC. RBS is caused by mutations in the SMC3 acetyltransferase ESCO2, whereas mutations in the DNA helicase DDX11 lead to WABS. We found that WABS-derived cells predominantly rely on ESCO2, not ESCO1, for residual SCC, growth and survival. Reciprocally, RBS-derived cells depend on DDX11 to maintain low levels of SCC. Synthetic lethality between DDX11 and ESCO2 correlated with a prolonged delay in mitosis, and was rescued by knockdown of the cohesin remover WAPL. Rescue experiments using human or mouse cDNAs revealed that DDX11, ESCO1 and ESCO2 act on different but related aspects of SCC establishment. Furthermore, a DNA binding DDX11 mutant failed to correct SCC in WABS cells and DDX11 deficiency reduced replication fork speed. We propose that DDX11, ESCO1 and ESCO2 control different fractions of cohesin that are spatially and mechanistically separated.

## Introduction

Sister chromatid cohesion (SCC) is mediated by cohesin, a presumed DNA-entrapping ring formed by structural maintenance of chromosome 1 and 3 (SMC1 and SMC3), RAD21 and SA1/2. The loader complex MAU2-NIPBL loads DNA into cohesin rings [1–3], whereas it can be released by the cohesin remover WAPL [4]. During DNA replication, stable cohesion is established in a process involving SMC3 acetylation by ESCO1 and ESCO2, which leads to the recruitment of Sororin and subsequent inhibition of WAPL activity [5–7]. The resulting SCC facilitates proper chromosome bi-orientation and equal distribution of genetic material during mitosis. Prior to chromatid separation in anaphase, cohesin needs to be removed, which happens in two rounds and via two distinct pathways [8, 9]. First, the prophase pathway promotes removal of cohesins from chromosome arms by WAPL, in a process involving multiple phosphorylations that restore WAPL activity [10]. Centromere cohesins are protected from the prophase pathway by SGOL1, which recruits the PP2A phosphatase to the centromeres [9, 11, 12]. In a separate step that occurs at the metaphase-to-anaphase transition, the remaining centromeric cohesins are removed by the protease Separase, which is activated by the Anaphase-Promoting Complex/Cyclosome (APC/C) and cleaves the RAD21 subunit [13]. In addition to its role in sister chromatid cohesion, the capacity of cohesin to entrap DNA also allows it to regulate gene transcription [14–16] and promote ribosome biogenesis [17–19].

Mutations in cohesin components or regulators result in a cluster of syndromes called cohesinopathies, characterized by diverse clinical abnormalities including growth retardation, intellectual disability, microcephaly and congenital abnormalities. Four cohesinopathies have been described thus far. Cornelia de Lange syndrome (CdLS) results from autosomal dominant or X-linked mutations in NIPBL, SMC1A, SMC3, RAD21 or HDAC8 [20–26]. Roberts Syndrome (RBS, also called SC phocomelia syndrome) is caused by bi-allelic mutations in ESCO2 [27]. Warsaw Breakage Syndrome (WABS) results from bi-allelic mutations in the DNA helicase DDX11 [28]. Chronic Atrial and Intestinal Dysrhythmia (CAID) syndrome was described in a patient with homozygous missense mutations in SGOL1 [29]. CdLS cells exhibit no obvious defects in SCC [30], and the clinical symptoms are thought to originate from deregulated gene expression (reviewed in [31–33]). By contrast, metaphases derived from RBS, WABS and CAID patient cells show severe cohesion loss [27–29]. The clinical symptoms of these syndromes are likely to originate from a combination of transcriptional defects and reduced progenitor cell proliferation.

ESCO1 and ESCO2, the vertebrate orthologues of yeast Eco1, share a conserved C-terminus that contains a zinc finger motif and an acetyltransferase domain, whereas no similarity is found in the N-terminus [34]. ESCO2 deficiency is embryonic lethal in mice, indicating that ESCO2 functions non-redundantly with ESCO1 [35]. RBS patient derived cells show defective centromere cohesion [36], in line with the observation that ESCO2 localizes to pericentric heterochromatin [35]. ESCO2 expression peaks during S-phase and is subsequently reduced by proteasomal degradation [35, 36] indicating that its prime function is to mediate SCC in the context of DNA replication. In budding yeast, Eco1 is reported to be recruited to the replication fork by replication factor PCNA [37] and in human cells, ESCO2 was found to interact with MCM components [38, 39], supporting a role for ESCO2 in replication-coupled cohesion. Decreased SMC3 acetylation in human cells reduces replication fork speed [40]. The authors proposed that the primary role of SMC3 acetylation would be to switch cohesin from a conformation that obstructs replication forks to a more open structure that allows fork progression [40]. Unlike ESCO2, ESCO1 is expressed during the whole cell cycle and has been reported to acetylate SMC3 independent of DNA replication, suggesting that ESCO1 also regulates non-canonical roles of cohesin [41]. Nevertheless, ESCO1 knockdown was also found to cause cohesion loss in Hela cells [34, 41] and DT40 chicken cells [42]. A different study reported no effect of ESCO1 knockdown or CRISPR mediated knockout on cohesion in Hela cells, and the authors proposed a model in which ESCO1 facilitates structural cohesion rather than replicative cohesion, thereby indirectly reinforcing cohesion that was established by ESCO2 [43].

DDX11 belongs to a group of ATP-dependent, super-family 2 (SF2) DNA helicases with an iron-sulfur cluster (Fe-S) domain [44]. It is specialized in unwinding certain DNA structures that contain a 5’-single stranded region, including forked duplexes, 5’-flap duplexes and anti-parallel G-quadruplexes (reviewed in [45]). This may be particularly relevant in the context of a DNA replication fork, where potentially long stretches of single stranded DNA can form secondary structures. Indeed, DDX11 and its yeast orthologue Chl1 have been shown to interact with multiple replication factors, such as the sliding clamp PCNA, its loader Replication Factor C complex (RFC), the 5’-flap endonuclease FEN1, the fork protection complex (FPC) component Timeless and with CTF4, which couples the MCM helicase to DNA polymerases [46–50]. How DDX11 deficiency results in cohesion loss is not entirely understood. The helicase-dependent resolution of complex secondary DNA structures that are formed particularly in the lagging strand may be required for successful sister chromatid entrapment, but helicase-independent roles in cohesin loading have also been proposed [50, 51]. Interestingly, Eco1 and Chl1 genetically interact [52–54] and synthetic lethality between DDX11 and ESCO2 was also reported in chicken DT40 cells [55].

Here we report synthetic lethality between ESCO2 and DDX11 in different human cell lines. Lethality is accompanied by aggravated cohesion loss in metaphase spreads. Reinforcing arm cohesion by WAPL knockdown rescues synthetic lethality, indicating that lethality results from detrimental loss of SCC. ESCO1 and ESCO2 appear to have both overlapping and non-overlapping roles in SCC, which are conserved between mice and men. We find that a DNA binding mutant of DDX11 cannot correct SCC in WABS cells and that DDX11 deficiency causes reduced replication fork speed. We propose that DDX11 and ESCO2 have functions in SCC that are both spatially and mechanistically distinct.

## Materials and methods

### Cell culture and construction of cell lines

RPE1-hTERT cells (American tissue collection) and SV40 transformed fibroblasts, including WABS [28], RBS [36] and LN9SV control [71], were cultured in Dulbecco’s Modified Eagles Medium (DMEM, Gibco), supplemented with 10% FCS, 1 mM sodium pyruvate and antibiotics.

CRISPR-Cas9 was used to construct DDX11 and ESCO2 knockouts in RPE1 cells. The generation of RPE1-hTERT_TetOn-Cas9_TP53KO cells is also described in a currently submitted manuscript [68]. Briefly, Cas9 cDNA was cloned into the pLVX-Tre3G plasmid (Clontech) and lentiviral Tre3G-Cas9 and Tet3G particles were produced in HEK293T cells using the Lenti-X HT packaging system (Clontech). Transduced cells were selected with 10 μg/mL puromycin and 400 μg/mL G418. Cells were treated with 100 ng/mL doxycycline (Sigma-Aldrich) to induce Cas9 expression and transfected with 10 nM synthetic crRNA and tracrRNA (Dharmacon or IDT) using RNAiMAX (Invitrogen). The following crRNA sequences were used: TP53 (CCATTGTTCAATATCGTCCG), DDX11-specific (GGCTGGTCTCCCTTGGCTCC), ESCO2 (TAAGTGGTACCTCAATCCAC). Single clones were assessed by Sanger sequencing using the following primers: TP53-Fw (GAGACCTGTGGGAAGCGAAA, TP53-Rv GCTGCCCTGGTAGGTTTTCT), DDX11-Fw (AACAACCCACCCTCCCCAAG), DDX11-Rv (TGCCTCACTCTCTCCAGACC), ESCO2-Fw (ATCAAAAAGGTAGAAGATGTCCAAGAAC), ESCO2-Rv (GCCTGTTTGATGGGTTCTGC).

### Proliferation assays

Adherent cells, the IncuCyte Zoom instrument (Essen Bioscience) was used. RPE1 cells (1500 / well) and fibroblasts (3000 / well) were seeded in 96-wells plates and imaged every 4 hours with a 10x objective. IncuCyte software was used to quantify confluence from four non-overlapping bright field images per well, for at least three replicate wells. Doubling time was calculated for the period required to grow from approximately 30% to 70% confluence, using the formula doubling time (h) = required time (h) * log(2) / (log(confluence endpoint(%))–log(confluence starting point(%))).

### siRNA transfections

For knockdown experiments, 25 nM siRNA (Dharmacon) was transfected using RNAiMAX (Invitrogen). Sequences: non-targeting siRNA UAAGGCUAUGAAGAGAUAC, siDDX11 GCAGAGCUGUACCGGGUUU, CGGCAGAACCUUUGUGUAA, GAGGAAGAACACAUAACUA, UGUUCAAGGUGCAGCGAUA, siESCO1 GGACAAAGCUACAUGAUAG, siESCO2 CAAAAUCGAGUGAUCUAUA GAGAGUAGUUGGGUGUUUA, AAUCAAGGCUCACCAUUUA, GAAGAAAGAACGUGUAGUA, siUBB CCCAGUGACACCAUCGAAA, GACCAUCACUCUGGAGGUG, GUAUGCAGAUCUUCGUGAA, GCCGUACUCUUUCUGACUA.

### Proliferation assays

Proliferation assays were performed in 96-wells plates. Cells were counted and seeded in at least triplicates in a total volume of 100 μl medium. Optimized cell densities were: WABS cells 3,000/well, RBS cells 4,000/well, RPE1 cells 1500/well. For WABS and RBS cells, cells were incubated with 10 μl CellTiter-Blue reagent (Promega) for 2–4 h and fluorescence (560_Ex_/590_Em_) was measured in a microplate reader (TriStar LB 941, Berthold Technologies). To monitor cell growth of RPE1 cells, the IncuCyte Zoom instrument (Essen Bioscience) was used. Cells were imaged every 4h with default software settings and a 10x objective. The IncuCyte software was used to quantify confluence from four non-overlapping bright field images.

### qRT-PCR

Total RNA was extracted with the High Pure Isolation Kit (Roche) and cDNA was prepared with the iScript cDNA Synthesis Kit (Biorad). Quantitative reverse transcription polymerase chain reaction (qRT-PCR) was performed using SYBR Green (Roche) on a LightCycler 480 (Roche). Levels were normalized to the geometric mean of at least two housekeeping genes. Primer sequences:

hDDX11-Fw AACCTGTTCAAGGTGCAGCGATAC, hDDX11-Rv GAGAAGCTGGTCGCAGGGT

mDDX11-Fw TTGTGGCTGTTTTGGGAGGTAATG, mDDX11-Rv CACCTGGCTCTGAAAGAGAAAGTC

h/mESCO1-Fw CCTGGTGCTGCTCAACATT, h/mESCO1-Rv CAGGAGTGGGATCTGAGAAAGC

m/hESCO2-Fw ATCAAAAAGGTAGAAGATGTCCAAGAAC, m/hESCO2-Rs GCCTGTTTGATGGGTTCTGC

HPRT1-Fw TGACACTGGGAAAACAATGCA, HPRT-Rv GGTCCTTTTCACCAGCAAGCT

TBP-Fw TGCACAGGAGCCAAGAGTGAA, TBP-Rv CACATCACAGCTCCCCACCA

B2M-Fw ATGAGTATGCCTGCCGTGTGA, B2M-Rv GGCATCTTCAAACCTCCATG

### Immunoblotting

Cells were lysed in lysis buffer (50 mM Tris-HCl pH 7.4, 150 mM NaCl, 1% Triton X-100) with protease- and phosphatase inhibitors (Roche). For DNA-bound protein fractions, cells were lysed in lysis buffer for 10 min and centrifuged at 1300 g for 10 min. The pellet was subsequently lysed in lysis buffer containing 5 units/μL benzonase nuclease (Sigma) for 1 h and centrifuged at maximum speed for 5 min. Proteins were separated by 3–8% or 8–16% SDS-PAGE (NU-PAGE or BioRad) and transferred to immobilon-P membranes (Millipore). Membranes were blocked in 5% dry milk in TBST-T (10 mM Tris-HCl pH 7.4, 150 mM NaCl, 0.04% Tween-20), incubated with primary and peroxidase-conjugated secondary antibodies (DAKO Glostrup, Denmark) and bands were visualized by chemoluminescence (Amersham). Antibodies used for detection are mouse anti-DDX11 (B01P, Abnova), mouse anti-α-tubulin (B-5-1-2, Santa Cruz #sc-23948), mouse anti-vinculin (H-10, Santa Cruz sc-25336), guinea pig anti-ESCO2 [36], mouse anti-ESCO1 (gift from JM Peters), rabbit anti-PARP (9542, Cell signaling), AcSM3 (gift from K Shirahige), rabbit anti-SMC3 (A300-060A, Bethyl), rabbit anti-WAPL (A300-268, Bethyl), mouse anti-vinculin (H-10, sc-25336, Santa Cruz).

### Flow cytometry

Cells were harvested and washed in PBS and fixed in ice-cold 70% EtOH. For mitosis detection, cells were incubated with rabbit anti-pS10-Histone H3 (Millipore) for 1 h and with Alexa Fluor 488 goat-anti-rabbit (Invitrogen) for 30 min. Cells were washed and resuspended in PBS with 1:10 PI/RNase staining buffer (BD Biosciences) and analyzed by flow cytometry on a BD FACSCalibur (BD Biosciences). Cell cycle analysis was conducted with BD CellQuest software (BD Biosciences).

### Analysis of cohesion defects

Cells were incubated with 200 ng/mL demecolcin (Sigma-Aldrich) for 20 minutes. Cells were harvested, resuspended in 0.075 M KCl for 20 minutes and fixed in methanol/acetic acid (3:1). Cells were washed in fixative three times, dropped onto glass slides and stained with 5% Giemsa (Merck). Cohesion defects were counted in 25 metaphases per slide on two coded slides per condition.

## Results

### Synthetic lethality of DDX11 and ESCO2 is conserved in different human cell lines

**Fig 1:**
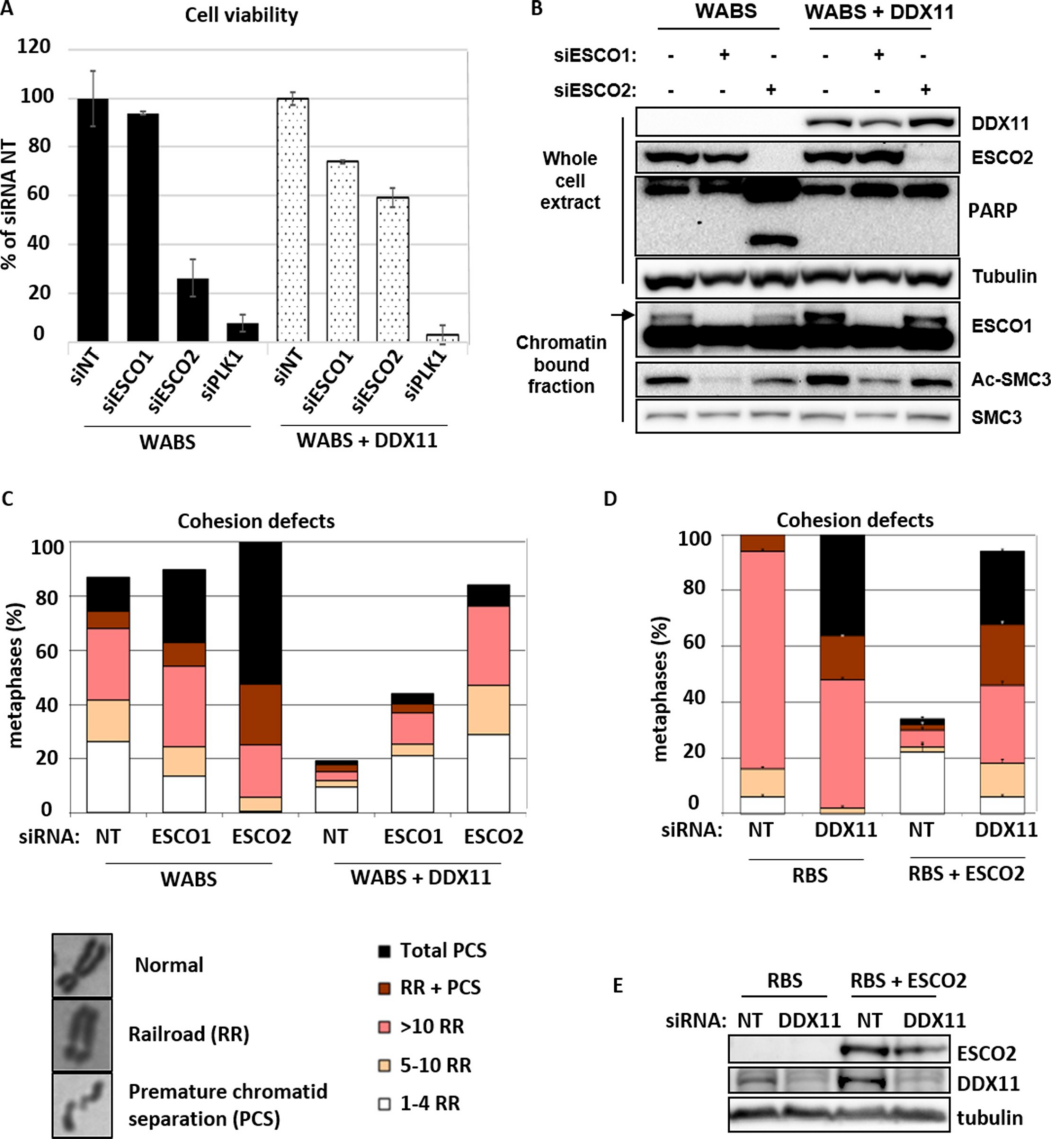
Synthetic lethality of DDX11 and ESCO2 is conserved in fibroblasts of WABS and RBS patients. (A) WABS fibroblasts and corrected cells were transfected with the indicated siRNAs and cell viability was analyzed after four days, using a cell-titer blue assay. (B) Cells were transfected with indicated siRNAs and analyzed by western blot after three days. WCE, whole cell extract. (C) Cohesion defects were quantified in metaphases spreads of cells transfected as in B. Examples of metaphase chromosomes with normal and railroad (RR) appearance, as well as premature chromatid separation (PCS), are shown. (D, E) RBS fibroblasts and corrected cells were transfected with the indicated siRNAs and assessed after three days by cohesion defect analysis and western blot.

We previously generated a unique isogenic cell line pair by functionally correcting fibroblasts derived from a WABS patient [56] and used these in a genome-wide siRNA screen to search for genes that are synthetically lethal with mutant DDX11 [57]. ESCO2 was one of the strongest hits as validated by deconvoluting the siRNA pool (S1 Fig). This confirms that the synthetic lethality between DDX11 and ESCO2, observed between yeast Chl1 and Eco1 [52–54] and chicken DDX11 and ESCO2 [55], is conserved in patient-derived cells. We used the same WABS cell line and its complemented counterpart to further validate these findings and also to examine the role of ESCO1 in this context. As expected, siRNA mediated ESCO2 knockdown strongly reduced the viability of WABS cells, but not of WABS+DDX11 cells (Fig 1A). This lethality correlated with increased levels of caspase-dependent PARP cleavage, reflecting apoptosis induction (Fig 1B). By contrast, knockdown of ESCO1 did not significantly affect cell growth in these cells, despite a substantial reduction of acetylated SMC3 which is the main target of ESCO1/ESCO2 acetyltransferases (Fig 1B). This suggests that ESCO1 mediated SMC3 acetylation contributes to non-cohesive or non-mitotic roles of the cohesion complex.

**Fig 2:**
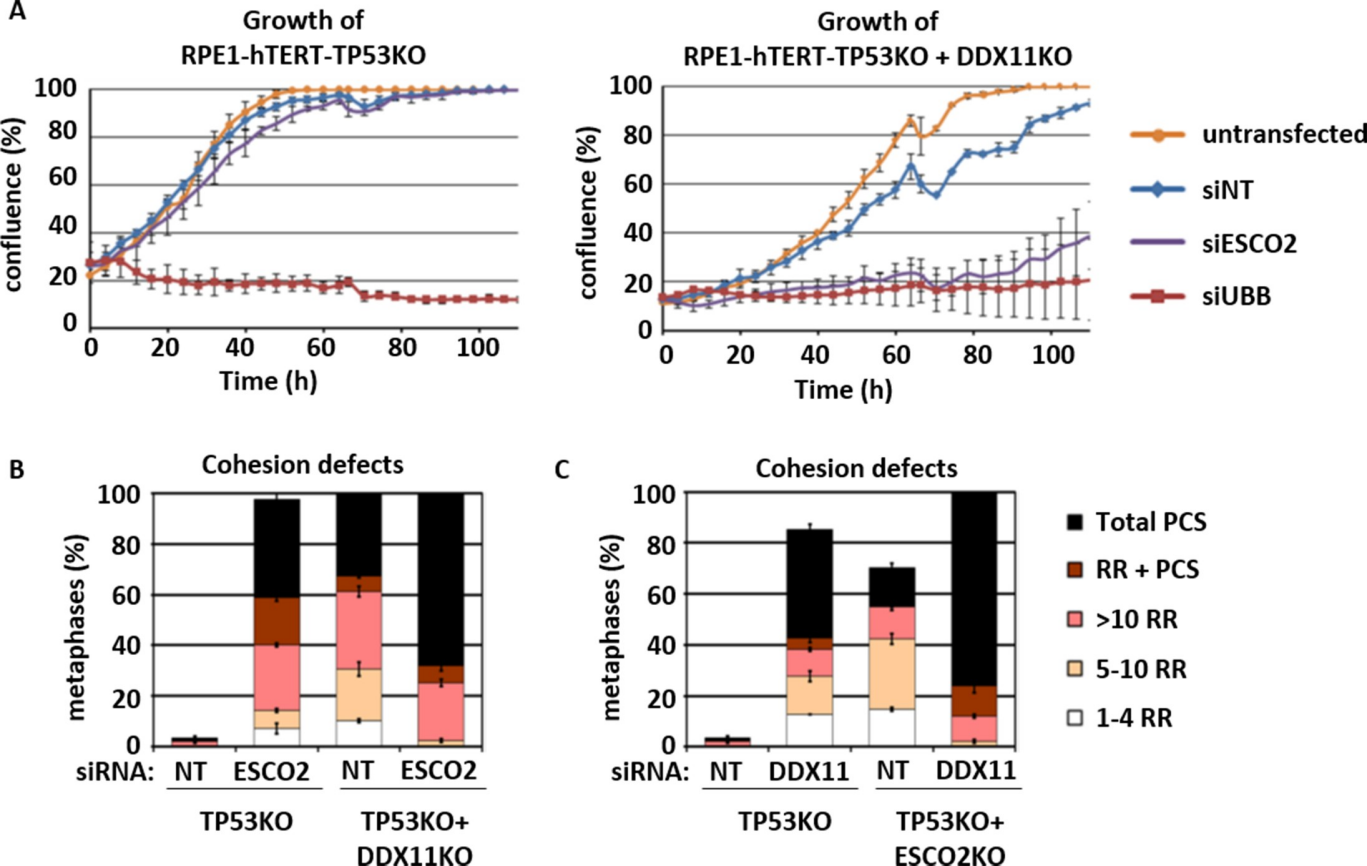
Synthetic lethality of DDX11 and ESCO2 in RPE1 cells. (A) CRISPR-Cas9 was used to knockout TP53 and DDX11 in RPE1-hTERT cells. The resulting isogenic cell lines were transfected with indicated siRNAs and proliferation was assessed using the IncuCyte. (B) Cells were transfected as in A and analyzed for cohesion defects. (C) CRISPR-Cas9 was used to disrupt the ESCO2 gene in RPE1-hTERT-TP53KO cells. Cells were transfected with indicated siRNAs and analyzed for cohesion defects.

In agreement with the effects of co-depleting DDX11 and ESCO1/2 on viability, we observed severely aggravated cohesion defects in WABS cells upon knockdown of ESCO2, whereas ESCO1 knockdown had a moderate effect (Fig 1C). Reversely, DDX11 knockdown exhibited a stronger effect on cohesion in RBS cells than in RBS cells corrected with ESCO2 cDNA (Fig 1D and 1E), although the effect was modest. For additional validation, we used CRISPR-Cas9 to create RPE1-TP53KO cells, and subsequently generated DDX11KOs and ESCO2KOs in this genetic background. Knockdown of ESCO2 specifically inhibited growth of RPE1-TP53KO-DDX11KO cells (Fig 2A) and caused increased levels of PCS (Fig 2B), showing that synthetic lethality cannot be rescued by TP53 loss. Similarly, cohesion defects were severely aggravated upon DDX11 knockdown in RPE1-TP53KO-ESCO2KO cells (Fig 2C).

**Fig 3:**
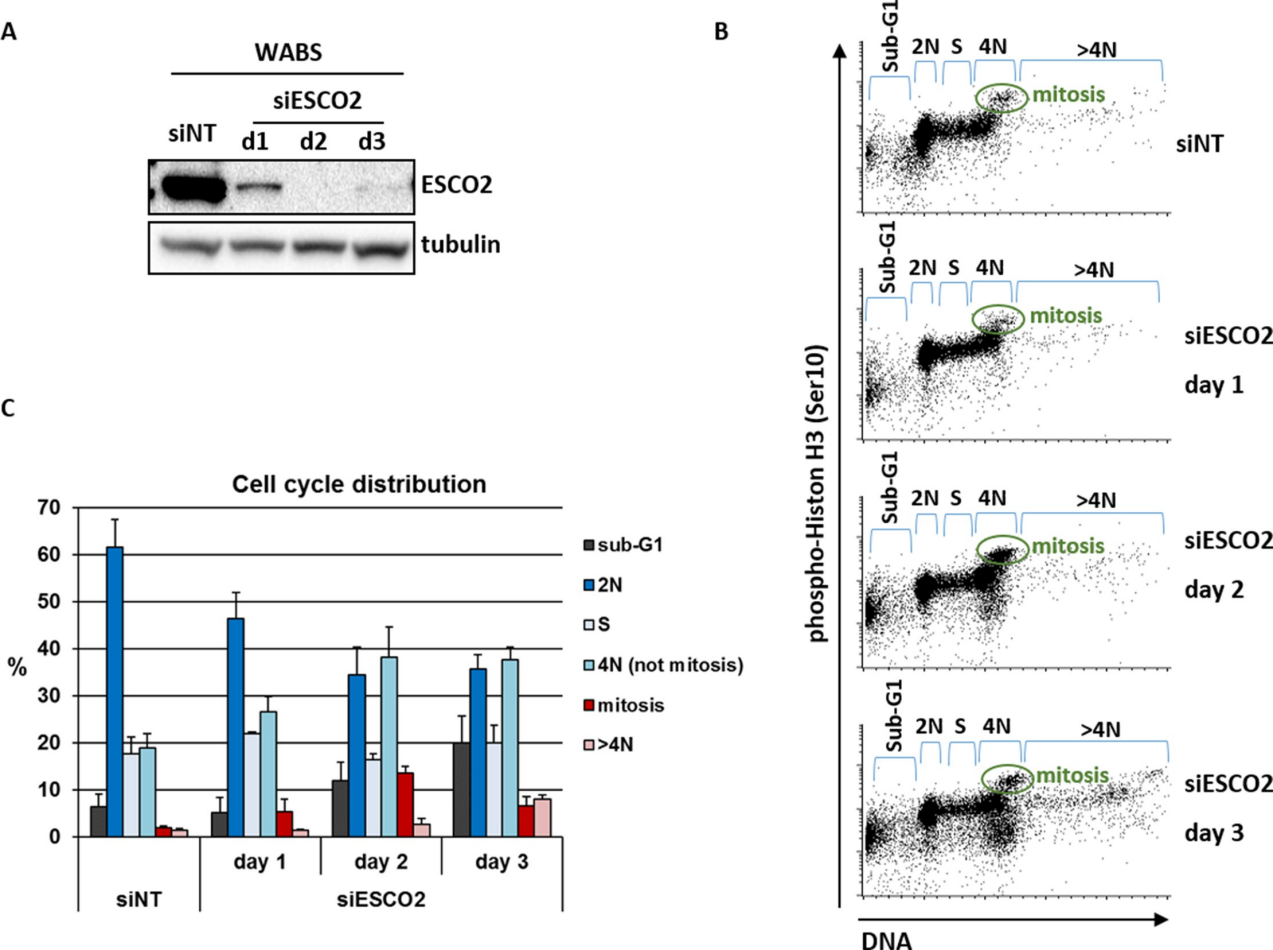
Induction of 4N fractions upon ESCO2 knockdown in WABS fibroblasts. WABS cells were transfected with the siESCO2, harvested at the indicated time points and analyzed by western blot (A) and flow cytometry (B). (C) Quantification of two independent experiments.

We further analyzed the effect of ESCO2 knockdown in WABS fibroblasts using flow cytometry, and found a clear induction of cells with a 4N DNA content (Fig 3A–3C). This includes both p-Histone H3 positive (mitotic cells) and p-Histone H3 negative cells (G2 cells, 4N G1 cells). This could in part reflect a G2 arrest, resulting from replicative stress or reduced DNA repair capacity. In addition, a small fraction of polyploid (>4N) cells appear at day 3. These findings probably reflect an extended metaphase duration via reactivation of the spindle assembly checkpoint that follows from reduced SCC and premature chromatid separation, as previously reported by us and others [57–59]. Eventually these arrested cells die in mitosis (as monitored by an increase in sub-G1 fraction), or may slip out of mitosis without cytokinesis. Cells that slip out of mitosis may subsequently continue to replicate, possibly contributing to the increased 4N fraction and leading to polyploidy.

### Synthetic lethality of DDX11 and ESCO2 is rescued by WAPL knockdown

**Fig 4:**
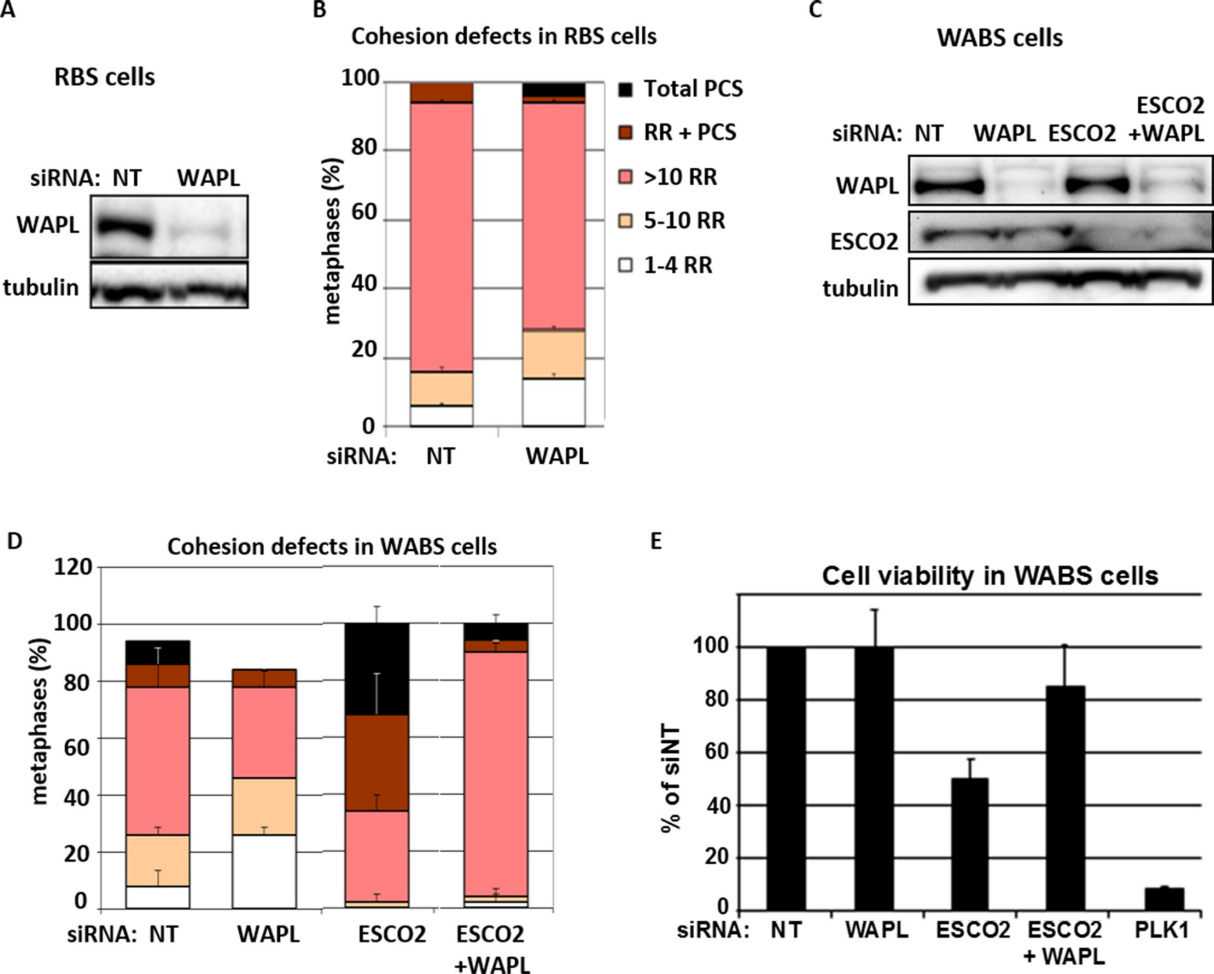
Restoring arm cohesion by WAPL knockdown rescues PCS and lethality. (A,B) RBS cells were transfected with siWAPL and harvested after three days for western blot and analysis of cohesion defects. (C-E) WABS cells were transfected with the indicated siRNAs and harvested after three days for western blot and analysis of SCC. Viability was assessed after four days with a cell-titer blue assay.

Next, we tested whether the SCC defects caused by single or double depletion of DDX11 and ESCO2 can be rescued by WAPL knockdown. WAPL plays a key role in removing cohesin rings from chromosome arms during the prophase pathway, so WAPL knockdown specifically causes hyper-cohesion on chromosome arms during metaphase. The cohesion defects in RBS cells could not be rescued by WAPL knockdown (Fig 4A and 4B). Since the WAPL-dependent prophase pathway only affects arm cohesion, this indicates that the effect of ESCO2 is predominantly manifested on centromere cohesion. In WABS cells, however, WAPL knockdown did prevent PCS and rescued both synthetic lethality and PCS by ESCO2 knockdown (Fig 4C–4E). The remarkable increase in railroad chromosomes in this triple knockdown condition can be explained by a further reduction of centromere cohesion in the absence of both DDX11 and ESCO2, as compared to single DDX11 depletion, combined with reinforcement of arm cohesion by WAPL knockdown.

In summary, co-depletion of DDX11 and ESCO2 results in severe cohesion loss and lethality, which are both rescued by reinforcing cohesion via WAPL knockdown. This indicates that ESCO2 and DDX11 critically facilitate SCC in partially separated contexts.

### ESCO1 and ESCO2 have separate functions in sister chromatid cohesion

**Fig 5:**
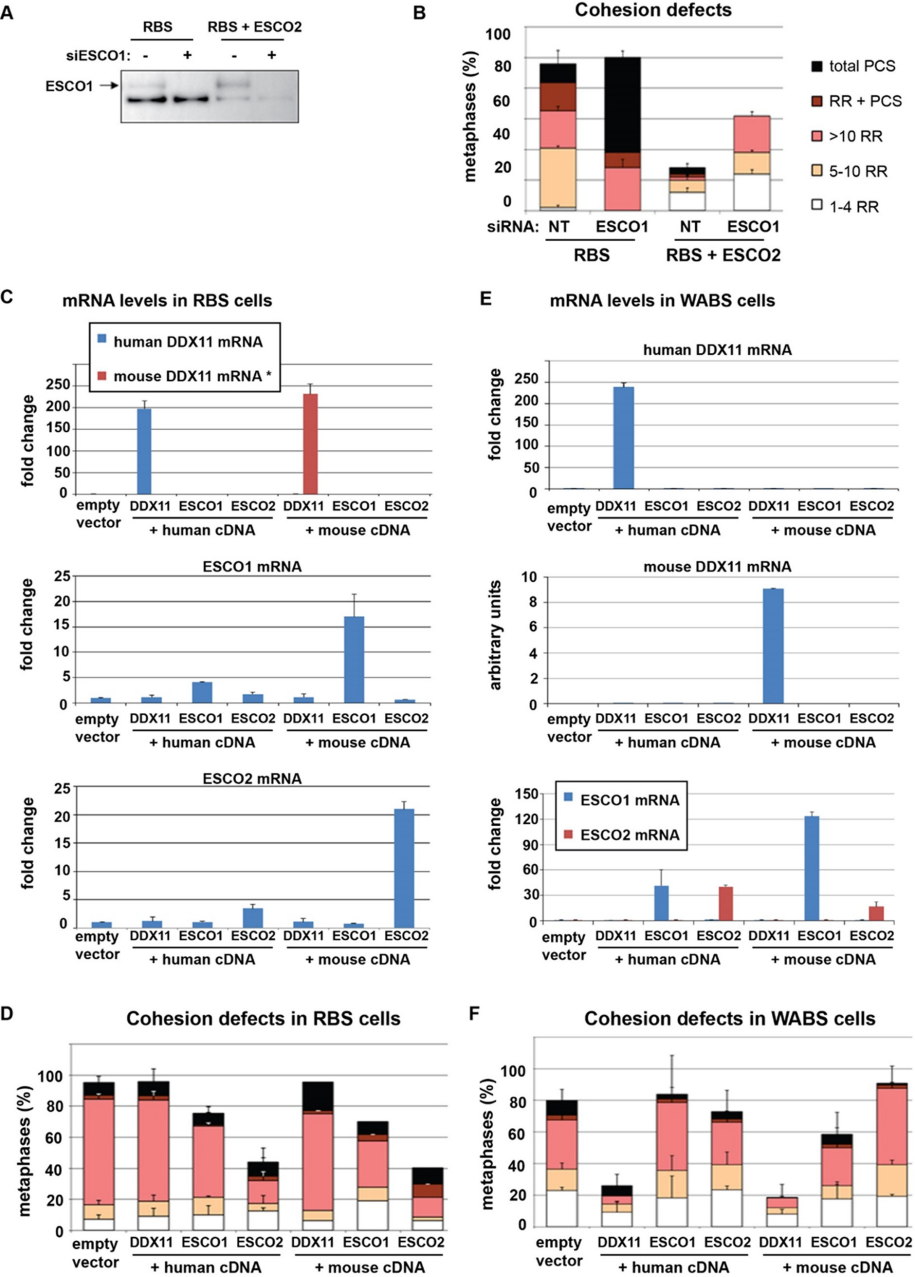
ESCO1, ESCO2 and DDX11 have separable functions in SCC. (A,B) RBS fibroblasts and corrected cells were transfected with siRNA targeting ESCO1 and harvested after three days for Western blot and SCC analysis. Mean and standard deviations of two technical replicates are shown. (C,D) RBS cells were transduced with lentiviral vectors expressing the indicated proteins and selected with puromycin. Overexpression was confirmed with qRT-PCR and cells were analyzed for cohesion defects. Mean and standard deviations of two independent experiments are shown. Fold changes were calculated relative to parental cells. Note that mouse DDX11 mRNA is not detected in human cells, so these values represent arbitrary units (*). DDX11 (E,F) WABS cells were transduced with lentiviral vectors expressing the indicated proteins and selected with puromycin. Overexpression was confirmed with qRT-PCR and cells were analyzed for cohesion defects. Mean and standard deviations of two independent experiments are shown. Fold changes were calculated relative to parental cells.

Since ESCO1 and ESCO2 both contribute to SMC3 acetylation, we set out to investigate the extent of their redundancy in SCC by manipulating their expression levels in RBS cells. Depletion of ESCO1 severely aggravated the cohesion defects in RBS cells and we also detected a small effect of ESCO1 depletion in RBS+ESCO2 cells (Fig 5A and 5B), suggesting that ESCO1 has a role in SCC that cannot be entirely compensated for by ESCO2.

Next, we investigated whether overexpression of ESCO1 or ESCO2 could rescue the cohesion defects in RBS cells. To assess possible species-specific effects, considering a lethal effect of ESCO1/2 or DDX11 deletion in mice [35, 60, 61], we also performed overexpression experiments using expression constructs of their mouse orthologues. Whereas cohesion in RBS cells could be restored similarly by either human or mouse ESCO2, the effect of ESCO1 was small, indicating that ESCO2 has a unique role in SCC that cannot be replaced by ESCO1 (Fig 5C and 5D). In line with the above described separate roles of ESCO2 and DDX11 in SCC, significant DDX11 overexpression completely failed to rescue the cohesion defects in RBS cells. Next, we overexpressed ESCO1, ESCO2 and DDX11 in WABS cells. Cohesion defects in WABS were similarly restored by cDNAs encoding either human or mouse orthologues of DDX11 (Fig 5E and 5F). Importantly, high levels of either ESCO1 or ESCO2 failed to compensate for DDX11 inactivation, indicating that cohesion loss resulting from impaired DDX11 function cannot be rescued by increased expression of SMC3 acetyltransferases. In conclusion, we find that DDX11, ESCO1, and ESCO2 have separable roles in SCC, which are conserved between human and mouse.

### DDX11-Q23A mutant fails to rescue sister chromatid cohesion

**Fig 6:**
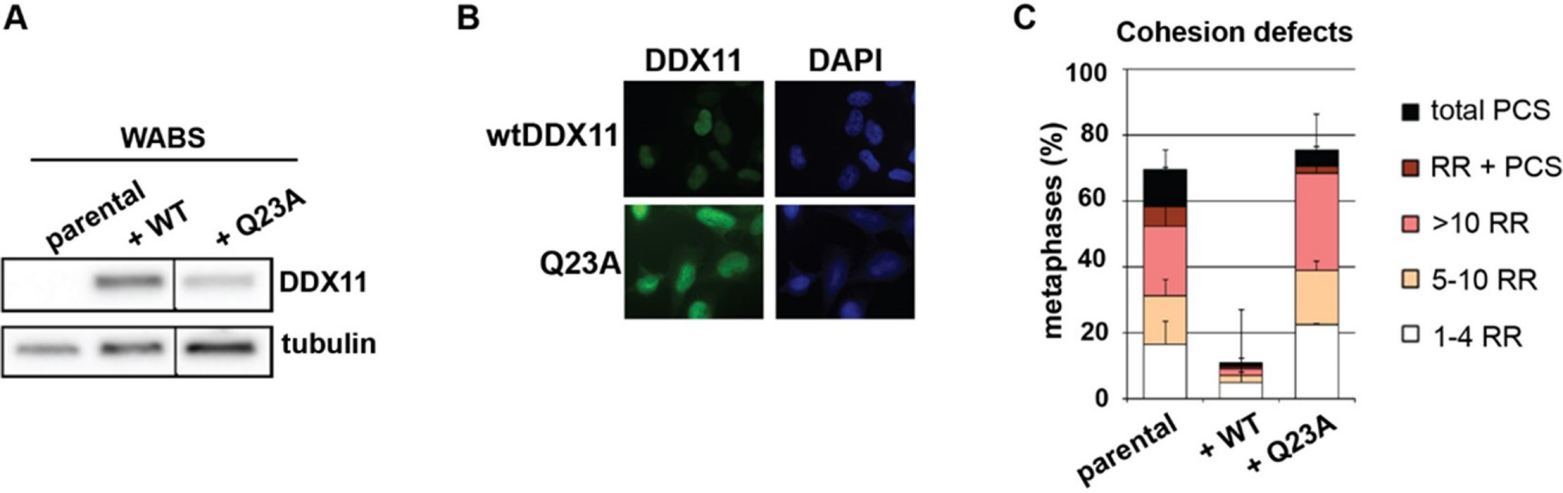
DDX11-Q23A mutant fails to rescue sister chromatid cohesion. (A) WABS fibroblasts were stably transfected with WT-DDX11 or the DNA binding mutant DDX11-Q23A, and assessed for protein levels using western blot. Lines within blots indicate positions where irrelevant lanes were removed. (B) The expression of ectopic DDX11 was assessed by immunofluorescence. (C) Cells were analyzed for cohesion defects. Mean and standard deviations of two independent experiments are shown.

Recent studies in different organisms reported conflicting results regarding whether or not the helicase function of DDX11/Chl1 is required for sister chromatid cohesion [50, 51, 55]. Therefore, we overexpressed the DNA-binding mutant DDX11-Q23A [62] in WABS cells and confirmed high nuclear DDX11 expression in the vast majority of cells (Fig 6A and 6B). We then assessed its ability to restore sister chromatid cohesion and find that whereas wtDDX11 rescues the cohesion loss in WABS fibroblasts, DDX11-Q23A shows no effect, suggesting that DDX11 helicase activity is required to promote or safeguard sister chromatid cohesion in human cells.

### DDX11 deficiency causes reduction of DNA replication fork speed and SMC3 acetylation

**Fig 7:**
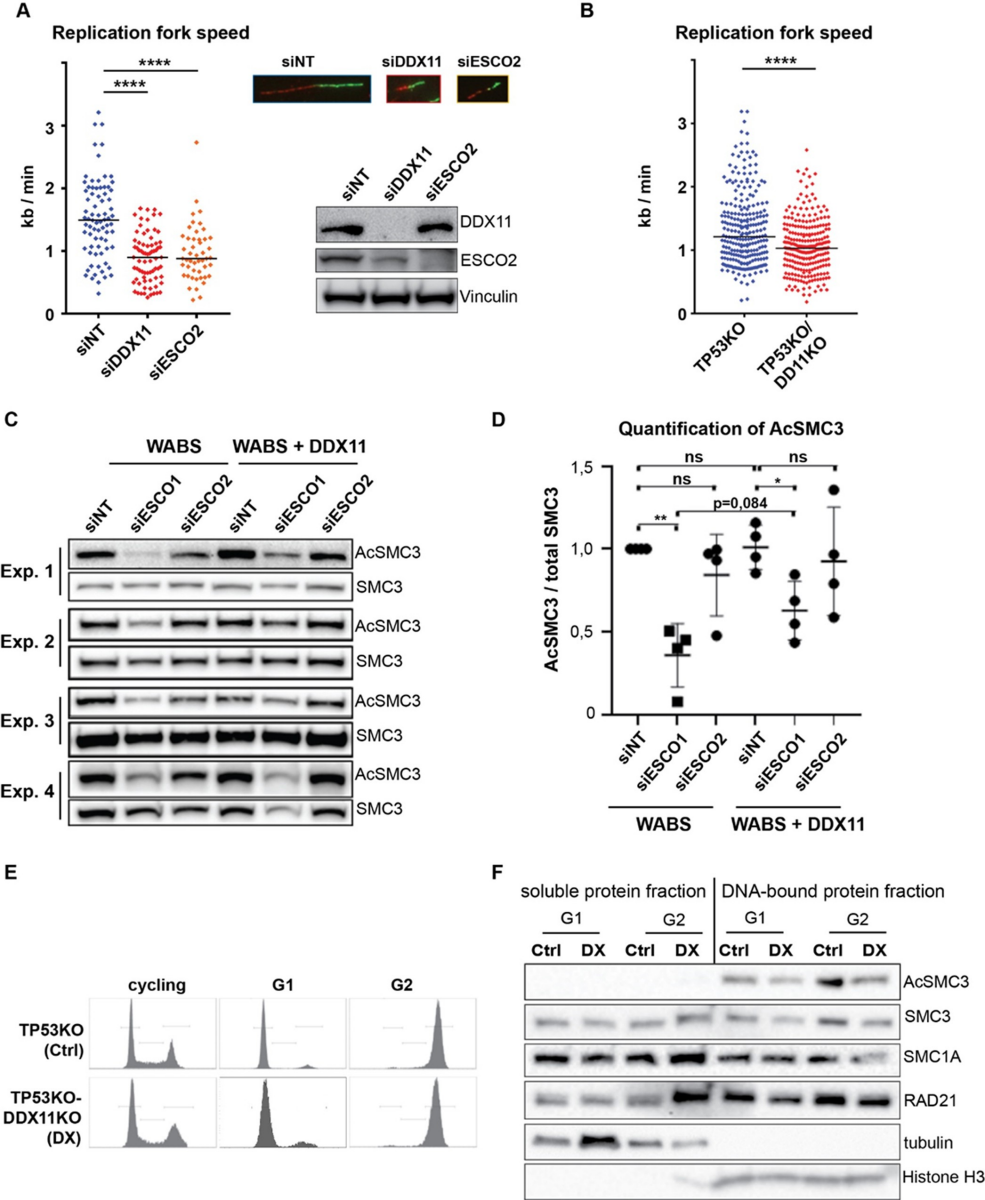
DDX11 deficiency slows replication fork speed and reduces SMC3 acetylation. (A) RPE1-hTERT cells were transfected with indicated siRNAs for two days and analyzed by western blot. Replication fork speed was assessed with a DNA fiber assay using a double labeling protocol. Black lines indicate the median. P-values were calculated by a non-parametric one-way ANOVA test. **** p<0,0001. Representative images of DNA fiber tracts are shown. (B) RPE1-TP53KO and RPE1-TP53KO-DDX1KO cells were analyzed with a DNA fiber assay. (C) WABS fibroblasts and corrected cells were transfected with the indicated siRNAs and analyzed by western blot after three days. Additional antibody incubations are provided in S2 Fig. (D) Quantification of AcSMC3 levels normalized to total SMC3, using image-lab software and. Mean and standard deviations are shown. P-values were calculated using a two-tailed t-test. * p<0,05; **<0,01; ns not significant. (E) RPE1-TP53KO and RPE1-TP53KO-DDX11KO cells were arrested in G1 using the Cdk4/6 inhibitor Palbociclib (20h, 10 μM) and in G2 using the Cdk1 inhibitor RO3306 (20h, 10 μM) and assessed by flow cytometry. (F) cells from (E) were lysed, and soluble and DNA-bound protein fractions were separated and analyzed by western blot. Ctrl, TP53KO cells; DX, TP53KO-DDX11KO cells.

The interaction of DDX11 with multiple replication fork components [46–50] suggests that it plays a role in cohesin loading in synchrony with DNA replication fork passage. To investigate whether DDX11 also promotes DNA replication itself, we performed DNA fiber assays. Indeed, we found that DDX11 knockdown reduces replication fork speed in RPE1-TERT cells, to levels comparable to those observed after ESCO2 depletion (Fig 7A and 7B). Based on our observations, we speculate that an important function of DDX11 is to resolve specific secondary DNA structures at the replication fork, thereby promoting replication fork progression and subsequent cohesin loading.

Impaired cohesin loading at replication forks in the absence of DDX11 helicase activity would be predicted to result in reduced cohesive effects of cohesin on DNA. Although we don’t observe an effect of DDX11 on SMC3 acetylation in WABS cells (Figs 7C, 7D and S2), it is possible that fluctuations are masked by SMC3 acetylation that occurs in the context of non-canonical or non-cell-cycle related cohesin activities (*e*.*g*. gene transcription). Indeed, when the ESCO1-dependent effects were eliminated by siRNA, DDX11 overexpression seemed to slightly increase SMC3 acetylation, but the effect was not significant (Fig 7D). To further examine this, we arrested RPE1-TP53KO-DDX11KO cells in G1 and G2 and compared the levels of SMC3 acetylation (Fig 7E and 7F). This revealed an increase in Ac-SMC3 in TP53KO cells in G2 as compared to G1 cells, whereas this increase was less pronounced in RPE1-TP53KO-DDX11KO cells. However, the effect was subtle and combined loss of DDX11 and ESCO2 had no synergistic impact on SMC3 acetylation (Fig 7D), despite severely aggravated cohesion loss. So, apart from a small, partially collaborative function between DDX11 and ESCO2 in promoting SMC3 acetylation, there clearly is also an Ac-SMC3-independent role of DDX11 in sister chromatid cohesion. This might relate to DNA replication fork stability or a role for DDX11 in second strand capture to promote cohesion, such as proposed by [63]. We propose that the DDX11 helicase promotes proper cohesin loading at replication forks, indirectly contributing to subsequent acetylation and stable sister chromatid cohesion.

## Discussion

In this study we provide evidence of synthetic lethality between ESCO2 and DDX11 in different human cell lines. Lethality correlated with prolonged mitosis and strongly aggravated loss of SCC, suggesting that mitotic arrest is triggered by premature chromatid separation and subsequent mitotic checkpoint activation, as we described before [57]. In line, we also observed signs of apoptosis induction and polyploidy. The synthetic lethality of DDX11 and ESCO2, as well as the failure of overexpression of one to compensate for loss of the other, indicates that ESCO2 and DDX11 act in different pathways leading to SCC establishment. Importantly, both lethality and cohesion loss were rescued by WAPL knockdown. This suggests that loss of only one of these proteins leads to a level of cohesion loss that can still be tolerated in most cells, but their combined loss causes near to complete cohesion loss, which is detrimental for cell viability.

The observed effects of WAPL knockdown suggest that the SCC resulting from the activity of these pathways is in part spatially separated at the chromosomes. An attractive explanation for the synthetic lethality between ESCO2 and DDX11 is that they largely function in spatially separated contexts. A previous study showed that ESCO2 loss particularly affects cohesion in pericentromeric regions [35], which is also illustrated by the large number of railroad chromosomes observed in metaphase spreads of RBS cells. It has to be noted that RPE1-ESCO2KO cells also exhibit considerable levels of PCS, indicating that the role of ESCO2 clearly is not restricted to establishing SCC at centromeres alone. Indeed, ESCO2 has been reported to bind PCNA and MCM complexes [37–39], and ESCO2 loss delays fork progression [40]. However, WAPL knockdown hardly rescues the cohesion loss in RBS cells, probably because inhibition of WAPL mainly reinforces arm cohesion by blocking the prophase pathway [4, 6, 64]. By contrast, WAPL knockdown partially rescues cohesion defects in DDX11 deficient cells, suggesting that DDX11 facilitates SCC on both chromosome arms and centromeres. This functional separation model receives particular support from our observation that in siESCO2 treated WABS cells, WAPL depletion only prevents PCS, but at the same time increases the amount of railroad chromosomes (Fig 3E).

The evolutionary divergence of yeast Eco1 into two vertebrate orthologues, ESCO1 and ESCO2, is accompanied by functional specialization and differential regulation of their protein levels. For example, ESCO1 is expressed during the whole cell cycle, whereas ESCO2 protein expression peaks in S-phase [35, 36]. This suggests that ESCO2 mainly fulfills the role of establishing SCC prior to regulated chromatid separation in mitosis, whereas ESCO1 directs cohesin’s non-canonical activities such as those related to gene transcription. Indeed, ESCO1 was reported to acetylate SMC3 in a replication-independent manner, via a unique interaction with PDS5 [41]. This might also explain why we observe a larger effect on SMC3 acetylation of ESCO1 knockdown as compared to ESCO2 knockdown. In line with earlier reports [34, 41, 42], we show here that ESCO1 also contributes to canonical SCC, albeit only to a small extent. Whereas we observed some compensating effects of overexpressing ESCO1 in RBS cells, both knockdown and overexpression studies indicated that ESCO1 and ESCO2 have largely non-overlapping roles in SCC. This might relate to location-specific effects on SCC of ESCO1 and ESCO2; ESCO2-dependent acetylation has a preference for centromeric cohesins, whereas ESCO1 might preferentially acetylate cohesin at chromosome arms. It will be interesting to further quantify the levels of cohesive cohesin on chromosome arms and centromeres in ESCO1 and ESCO2 deficient human cells. Importantly, we could not distinguish the activities of human or mouse versions of DDX11 or ESCO1/2. Therefore, the paradox that ESCO2 loss is embryonically lethal in mice [35], whereas most human RBS patients contain loss of function mutations in ESCO2 [65], can probably not be explained by differences in the mouse and human ESCO1 and ESCO2 coding sequences. Possibly, the typical acrocentric architecture of murine chromosomes makes them more vulnerable to SCC deprivation and/or ESCO2 deficiency.

Our data indicate that the helicase activity of DDX11 critically promotes SCC in human cells. DDX11 is thought to resolve certain complex secondary DNA structures including GC-rich regions and/or anti-parallel G-quadruplexes [66, 67]. The duplex or quadruplex resolving capacities of DDX11 helicases may contribute to SCC by facilitating loading of the second strand into cohesin rings, which was reported to require single stranded DNA [63]. Alternatively, secondary DNA structures that are normally substrates of DDX11 may be prone to breakage when DDX11 is absent, requiring repair and WAPL/PDS5-dependent cohesin removal to provide access of repair factors to the break site [68].

Both DDX11 [46–50] and ESCO2 [37–39] interact with multiple replication fork components, indicating that they promote cohesin loading and cohesion establishment in synchrony with DNA replication fork passage. Possibly, DDX11 acts upstream of ESCO1/2 to facilitate cohesin loading and subsequent SMC3 acetylation. However, this is difficult to reconcile with the severe synthetic lethality of DDX11 and ESCO2: loss of one factor would disrupt the pathway, whereas loss of the second factor would have a small additional effect. As an alternative for spatially separated activities, DDX11 and ESCO2 may participate in mechanistically distinct cohesion establishment pathways. An important notion could be that there seems to be no synergistic effect of DDX11 and ESCO2 co-depletion on SMC3 acetylation. Moreover, we find that increasing SMC3 acetylation by overexpressing ESCO1 or ESCO2 cannot rescue the cohesion defects in DDX11 deficient cells. This may suggest that DDX11 has an Ac-SMC3-independent role in cohesion establishment. Indeed, several reports proposed that there are two parallel pathways to facilitate SCC [52, 69, 70]. Genetic dissection in yeast suggested that loss of Chl1 specifically disrupts one such pathway, but leaves the other intact. In conclusion, we propose that DDX11, ESCO1 and ESCO2 control different fractions of cohesin that are spatially and mechanistically separated.

## Supporting information

S1 Fig Deconvolution of siRNAs targeting ESCO2. WABS fibroblasts and corrected cells were transfected with the indicated siRNAs and cell viability was analyzed after four days, using a cell-titer blue assay.
(TIF)

S2 Fig Effect of DDX11, ESCO1 and ESCO2 on SMC3 acetylation. Cells were transfected with indicated siRNAs and analyzed by Western blot.
(TIF)
