# Supplementary figures and images for: Non-redundant roles in sister chromatid cohesion of the DNA helicase DDX11 and the SMC3 acetyl transferases ESCO1 and ESCO2
Source: PLoS One. 2020 Jan 14;15(1):e0220348. doi: 10.1371/journal.pone.0220348 (PMC6959578; doi:10.1371/journal.pone.0220348)

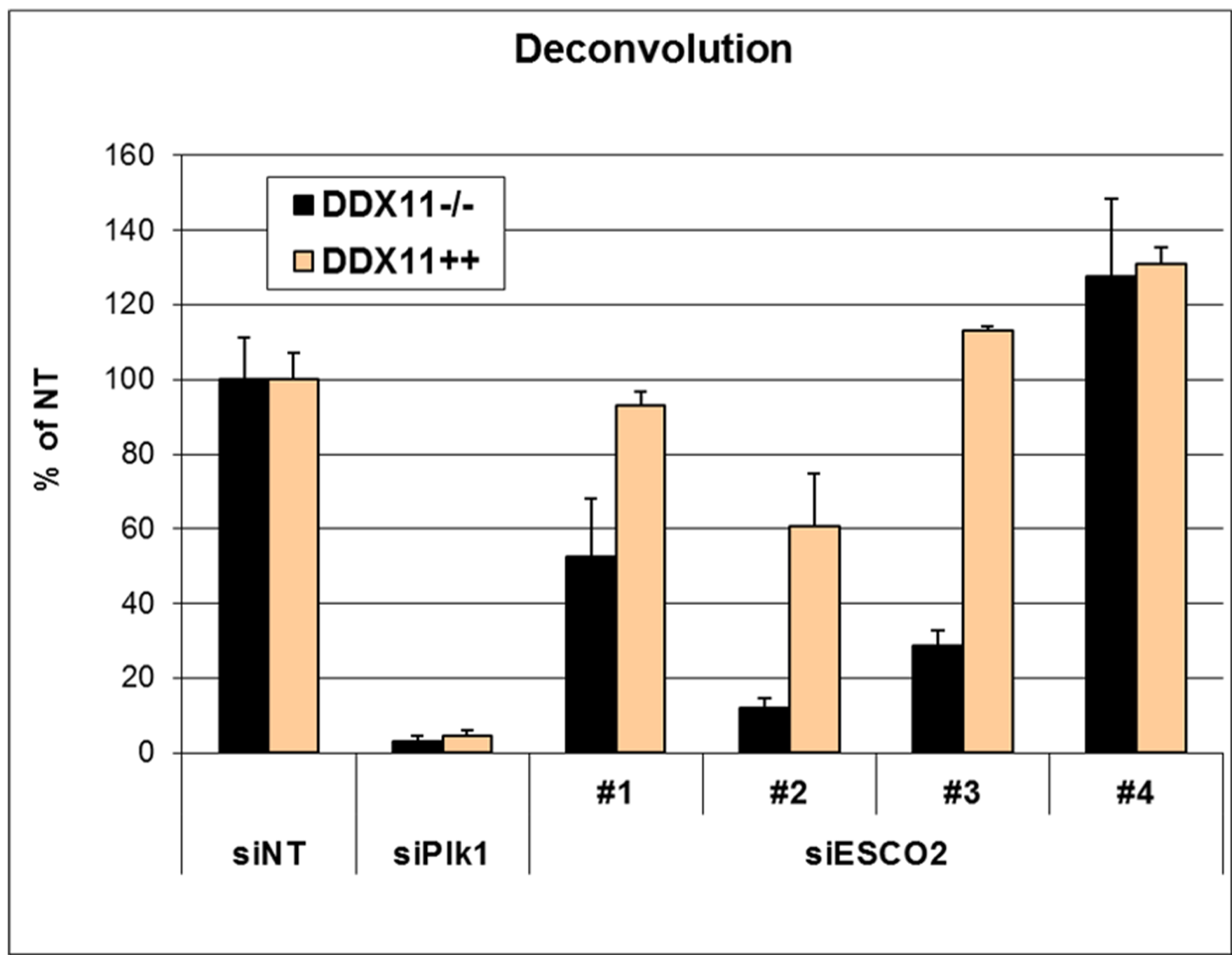

Supplement: S1 Fig — WABS fibroblasts and corrected cells were transfected with the indicated siRNAs and cell viability was analyzed after four days, using a cell-titer blue assay. (TIF) [file pone.0220348.s001.tif]

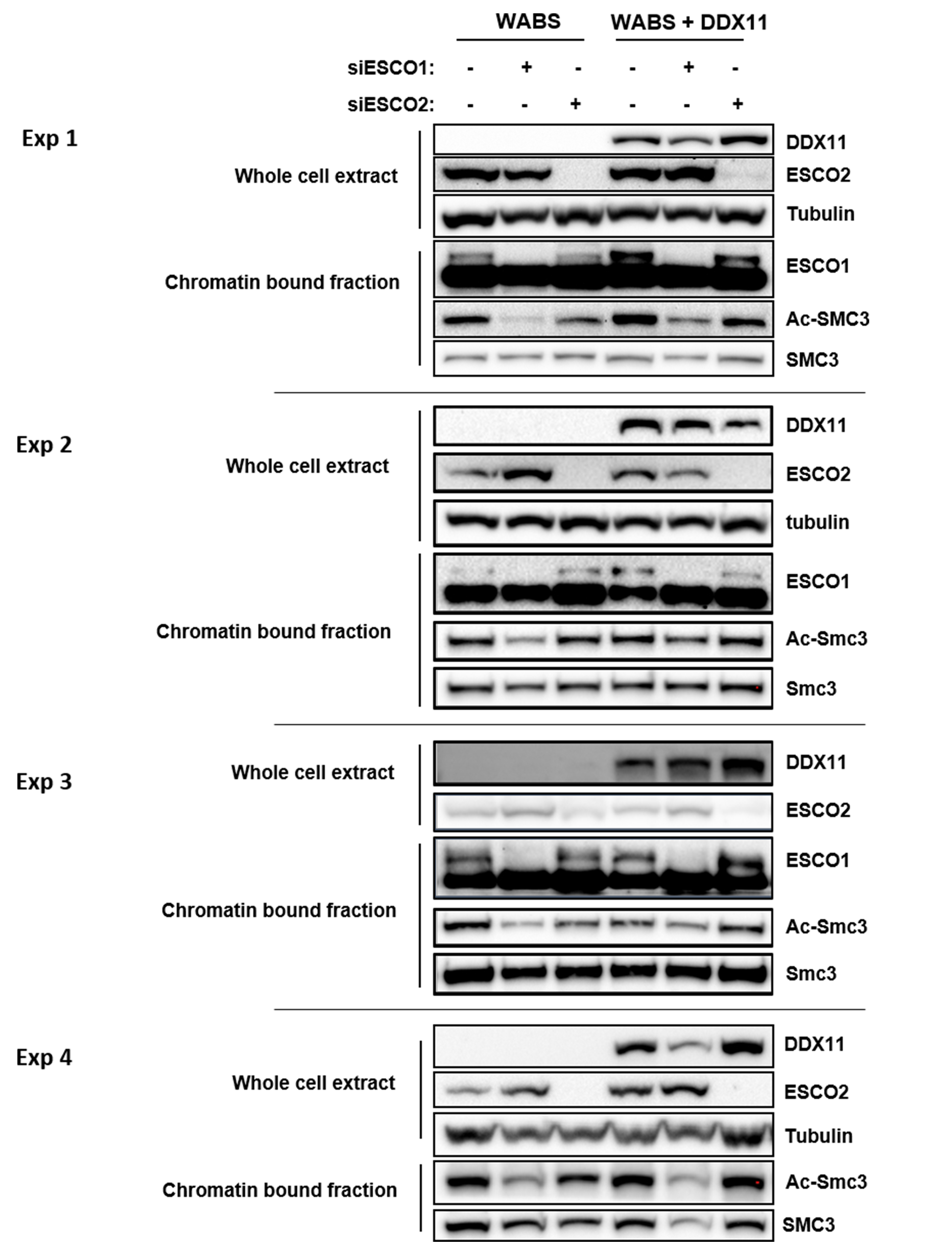

Supplement: S2 Fig — Cells were transfected with indicated siRNAs and analyzed by Western blot. (TIF) [file pone.0220348.s002.tif]
